# Point-of-care creatinine vs. central laboratory creatinine in the critically ill

**DOI:** 10.1016/j.ccrj.2024.07.002

**Published:** 2024-08-05

**Authors:** Kyle C. White, James McCullough, Kiran Shekar, Siva Senthuran, Kevin B. Laupland, Goce Dimeski, Ary Serpa-Neto, Rinaldo Bellomo, Queensland Critical Care Research Network (QCRCN)

**Affiliations:** a Intensive Care Unit, Princess Alexandra Hospital, Woolloongabba, Queensland, Australia; b Faculty of Medicine, University of Queensland, Brisbane, Queensland, Australia; c Queensland University of Technology (QUT), Brisbane, Queensland, Australia; d School of Medicine and Dentistry, Griffith University, Mount Gravatt, Queensland Australia; e Intensive Care Unit, Gold Coast University Hospital, Southport, Queensland, Australia; f Adult Intensive Care Services, The Prince Charles Hospital, Brisbane, Queensland, Australia; g College of Medicine and Dentistry, James Cook University, Townsville, Queensland, Australia; h Intensive Care Unit, Townsville Hospital, Townsville, Queensland, Australia; i Department of Intensive Care Services, Royal Brisbane and Women's Hospital, Brisbane, Queensland, Australia; j Department of Chemical Pathology, Pathology Queensland, Princess Alexandra Hospital, Woolloongabba, Queensland, Australia; k Australian and New Zealand Intensive Care Research Centre (ANZIC-RC), School of Public Health and Preventive Medicine, Monash University, Melbourne, Australia; l Department of Critical Care, University of Melbourne, Melbourne, Australia; m Department of Intensive Care, Royal Melbourne Hospital, Melbourne, Australia; n Department of Intensive Care, Austin Hospital, Heidelberg, Australia

**Keywords:** Critical care, Acute kidney injury, Creatinine, Point-of-care

## Abstract

**Objective:**

Frequent measurement of creatinine by point-of-care testing (POCT) may facilitate the earlier detection of acute kidney injury (AKI) in critically ill patients. However, no robust data exist to confirm its equivalence to central laboratory testing. We aimed to conduct a multicenter study to compare POCT with central laboratory creatinine (CrC) measurement.

**Design:**

Retrospective observational study, using hospital electronic medical records. Obtained paired point-of-care creatinine (CrP) from arterial blood gas machines and CrC.

**Setting:**

Four intensive care units in Queensland, Australia.

**Participants:**

Critically ill patients, where greater than 50% of POCT contained creatinine.

**Main outcome measures:**

Mean difference, bias, and limits of agreement between two methods, and biochemical confounders.

**Results:**

We studied 79,767 paired measurements in 19,118 patients, with a median Acute Physiology and Chronic Health Evaluation 3 score of 51. The mean CrC was 115.5 μmol/L (standard deviation: 100.2) compared to a CrP mean of 115 μmol/L (standard deviation: 100.7) (Pearson coefficient of 0.99). The mean difference between CrP and CrC was 0.49 μmol/L with 95% limits of agreement of −27 μmol/L and +28 μmol/L. Several biochemical variables were independently associated with the difference between tests (e.g., pH, potassium, lactate, glucose, and bilirubin), but their impact was small.

**Conclusion:**

In critically ill patients, measurement of creatinine by POCT yields clinically equivalent values to those obtained by central laboratory measurement and can be easily used for more frequent monitoring of kidney function in such patients. These findings open the door to the use of POCT for the earlier detection of acute kidney injury in critically ill patients.

## 1 Introduction

Acute kidney injury (AKI) is common in critically ill patients and is associated with significant morbidity and mortality.^1^^,^^2^ The diagnosis of AKI is based on urine output and creatinine.^3^ Traditionally, creatinine has been seen as a flawed and late biomarker of AKI because it increases only if approximately 50% of the glomerular filtration rate is lost and, therefore, cannot be used to trigger early protective interventions. However, another limitation to the value of creatinine as a biomarker in critically ill patients arises from the fact that it is typically measured in the central laboratory once or, at most, twice a day. It is possible that if creatinine could be easily measured by point-of-care testing (POCT) using arterial blood gas machine–based technology, some of its limitations as biomarkers may be attenuated.

Creatinine can now be measured by the point-of-care-based enzymatic amperometric method on a bench top using portable blood gas machines. However, multiple aspects of patient biochemistry and physiological derangements, which are present in patients admitted to the intensive care unit (ICU) may induce changes to both central laboratory and POCT results and make measurements less comparable.[4], [5], [6], [7] Moreover, in critically ill patients, the performance and relationship between POCT and central laboratory creatinine (CrC) measurements have only been tested in a small single-centre study.^8^ As rapid diagnosis of AKI is essential to management,^9^ and delayed detection of AKI has been associated with adverse patient outcomes;^10^^,^^11^ more frequent assessment of creatinine appears desirable. Such assessment could be enabled by POCT for creatinine if such POCT was shown to be robust and closely correlated with central laboratory measurements. Creatinine measurements could then be performed multiple times a day in combination with routine arterial blood gases. However, this technology is not widely practised.

Accordingly, we aimed to compare CrC and point-of-care creatinine (CrP) in a large, diverse population of critically ill patients from multiple ICUs.

## 2 Methods

### 2.1 Study design

We conducted a multicentre, retrospective cohort study of granular, routinely collected, electronic medical record (EMR)-based clinical data.

### 2.2 Study sites and patient identification

The study was conducted at four closed-model tertiary ICUs in Queensland, Australia. The study evaluated all adult patients admitted between January 1, 2015, and December 31, 2021. There were no clinical exclusion criteria. Patients were included in the study if greater than 50% of their arterial blood gas analyses included a creatinine measurement.

### 2.3 Data sources

Data were collected from all centres using eCritical MetaVision™ (iMDsoft, Boston, MA, USA) clinical information systems,[12], [13], [14], [15] the Australia and New Zealand Intensive Care Society (ANZICS) Adult Patient Database,[16], [17], [18], [19] the Queensland Health Admitted Patient Database Collection,[20], [21], [22] and the Queensland Births, Deaths, and Marriage Registry.^23^^,^^24^ Admission diagnoses were categorised to optimise data accuracy and interpretability (Supplementary Methods, Table S1). The Charlson-defined comorbidities and index were calculated from the International Classification of Diseases (ICD-10) codes (Supplementary Methods, Table S2).^25^^,^^26^ AKI was determined by comparing creatinine measurements to a calculated baseline creatinine. The baseline creatinine calculation was performed using a validated methodology.^27^^,^^28^ The severity of the AKI was defined as per the Kidney Disease: Improving Global Outcomes definition of AKI.^3^

### 2.4 Creatinine measurement

CrC was measured by Pathology Queensland laboratories on the general chemistry analysers at four hospitals. For all sites during the study period, the central lab measurement was based on the Jaffe rate method and performed on Beckman Coulter general chemistry analysers using the DxC800 model in two laboratories and the DxC600 model in the other two laboratories (Beckman Coulter, Brea, CA, USA). The Jaffe method, which has been used for more than 100 years, is the most prevalent method for measuring creatinine on laboratory analysers worldwide, primarily due to its low cost.^29^ The Jaffe method, also known as the alkaline picrate reaction, relies on the formation of a red-orange chromogen from the reaction of creatinine and picric acid in an alkaline medium, which can then be assessed by spectrophotometry.^30^ The absorbance of the chromogen is proportional to the creatinine concentration, according to the Beer–Lambert law.^31^

The CrP creatinine measurement was done using an enzymatic method in all four hospitals, as available in the Radiometer ABL800 analysers (Radiometer ABL800 FLEX, Copenhagen, Denmark) located in the ICU departments. The Radiometer ABL800 system uses creatinase and sarcosine oxidase enzymes in its sensor to generate hydrogen peroxidase from whole blood, which is converted into current by a platinum anode that is measured by the analyser.^32^

In this study, CrP and CrC measurements were assessed for near-simultaneous/paired collection. If the CrP occurred within 1 h on either side of the CrC, then the samples were considered paired. Any CrP measurements that were not paired were excluded from the analysis. The difference in creatinine measurements was calculated by subtracting CrP value CrC value, such that a positive difference meant that CrC was higher than CrP and a negative difference meant that CrP was higher than CrC.

### 2.5 Outcomes

The primary outcome was the correlation, mean difference, bias, and limits of agreement between the two methods used to determine creatinine. The secondary outcome was the identification of which biochemical factors were associated with the difference between creatinine values according to the two methods and the incidence of a difference in AKI stage between paired samples.

### 2.6 Statistical analysis

Descriptive statistics were expressed as frequencies and proportions for categorical variables and medians with interquartile ranges (IQRs) or means with standard deviations (SDs), depending on their parametric or nonparametric distribution. The correlation between creatinine measurements was assessed using Pearson's correlation coefficient. A Bland–Altman plot was constructed to compare the mean difference between the two creatinine measurement methods. A mixed-effects linear regression model, including the individual patient as a random effect, was developed to examine which biochemical variables were independently associated with the difference between CrC and CrP. The variables used for analysis were determined a priori. The results of the multivariable analysis were reported as coefficients with 95% confidence intervals (95% CIs). A sensitivity analysis was performed using one paired sample per patient to ensure repeated measurements within the same patient did not impact analysis (Supplementary Methods, Table S3). Given the large data set and multiple comparisons, a two-sided p-value of <0.01 was chosen to indicate statistical significance.^33^ Statistical analyses were performed using R v.4.0.3.

### 2.7 Ethical considerations

This study was approved by the Metro South Hospital and Health Service Human Research Ethics Committee (HREC/2022/QMS/82024) with an individual waiver of consent granted.

## 3 Results

### 3.1 Patient selection and characteristics

There were 51,988 adult admissions at the participating sites. Of these, 20,672 patient admission episodes achieved the threshold for arterial blood gas creatinine measurements, and 19,118 had at least one paired creatinine measurement. Among such patients, there were 79,767 paired creatinine measurement samples.

The patient cohort had a median age of 62 (IQR: 48–71) years and a median Charlson co-morbidity index of 3 (IQR: 1–5). The common reason for admission was cardiovascular (6762; 35%), followed by neurological (3134; 16%). At the time of admission, the median Acute Physiology and Chronic Health Evaluation 3 score was 51 (IQR: 38–68). Over half of patients were emergency admissions (10,622; 56%). During the ICU admission, most patients required ventilation (11,953; 63%) and vasopressors (10,024; 52%). The median ICU length of stay was 3 days (IQR: 2–5), and 1235 (6.5%) died in the ICU. The complete cohort description is presented in Table 1.

**Table 1:** Cohort characteristics, treatments, and outcomes.

Characteristic	N = 19,118^a^
Age (years)	62 (48, 71)
Female	7271 (38%)
Body mass index	28 (25, 31)
Charlson comorbidity index	3.00 (1.00, 5.00)
APACHE 3 score	51 (38, 68)
Admission diagnosis	
Cardiovascular	6762 (35%)
Gastrointestinal	2202 (12%)
Genitourinary	728 (3.8%)
Haematological	51 (0.3%)
Metabolic	915 (4.8%)
Neurological	3134 (16%)
Other	503 (2.6%)
Respiratory	1858 (9.7%)
Sepsis	1411 (7.4%)
Trauma	1554 (8.1%)
Admission circumstances	
Postelective surgery	8536 (45%)
Emergency admission	10,622 (56%)
Post-trauma	1554 (8.1%)
Post–cardiac arrest	838 (4.4%)
Interventions during ICU admission	
Any RRT	1334 (7.0%)
Any ventilation	11,953 (63%)
Any Vasopressors	10,024 (52%)
Outcomes	
ICU LOS (days)	3.0 (2.0, 5.0)
Hospital LOS (days)	10 (6, 18)
ICU Mortality	1235 (6.5%)
Day 90 mortality	1725 (9.0%)

Abbreviations: APACHE = Acute Physiology and Chronic Health Evaluation; ICU = intensive care unit; RRT = renal replacement therapy; LOS = length of stay; IQR = interquartile range.

a Median (IQR); n (%).

### 3.2 Correlation

**Fig. 1:**
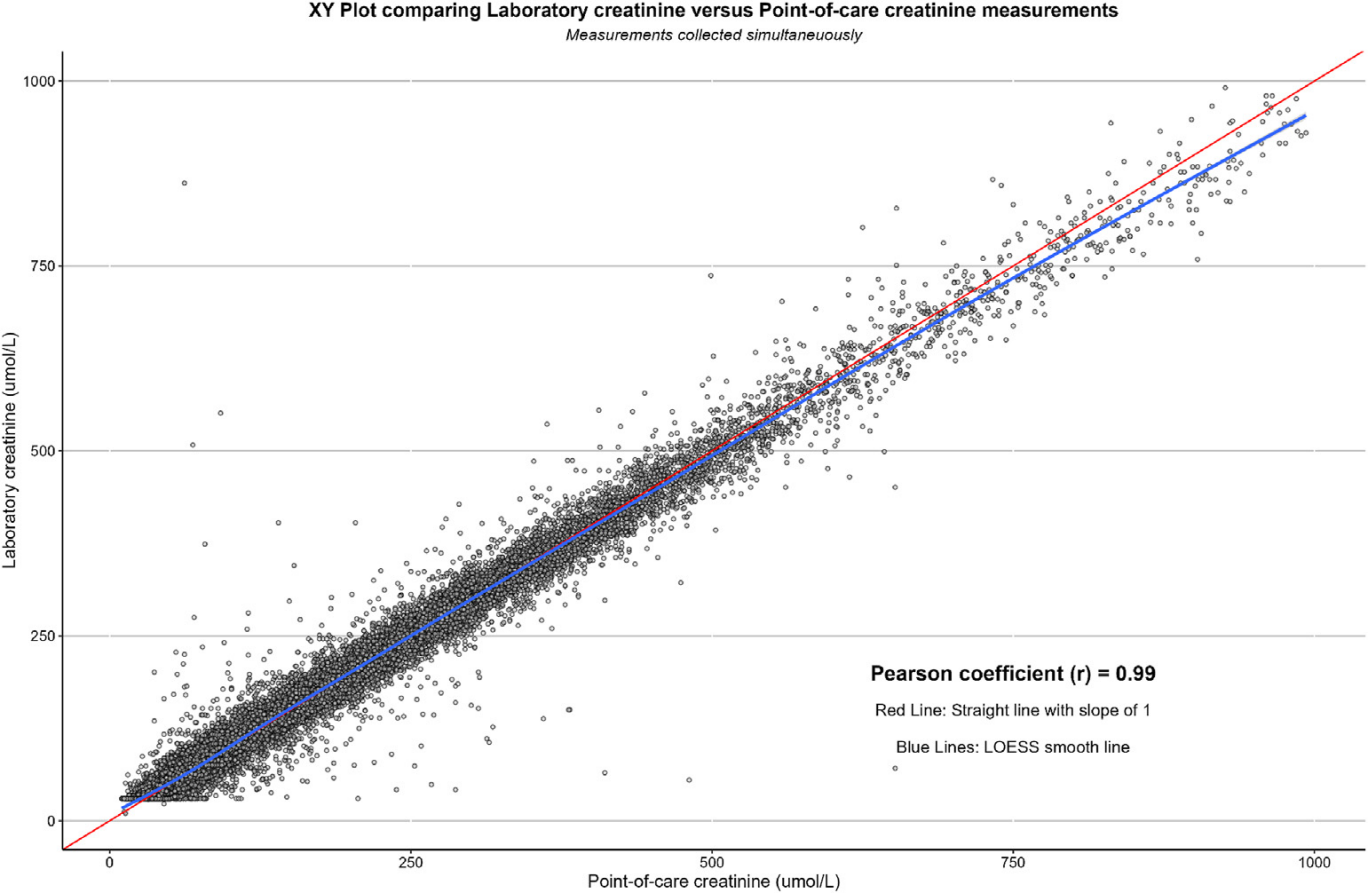
XY plot comparing laboratory creatinine versus point-of-care creatinine measurements.

**Fig. 2:**
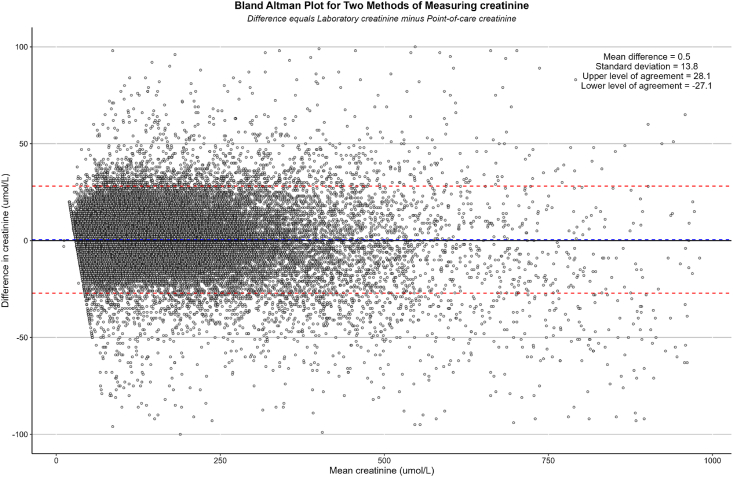
Bland–Altman plot for the two methods of measuring creatinine.

The mean CrC was 115.5 μmol/L (SD: 100.2), and the mean CrP was 115 μmol/L (SD: 100.7) (p < 0.001). The correlation between CrP and CrC is demonstrated in Fig. 1. The Pearson coefficient, *r*, was very high at 0.99. The Bland–Altman plot (Fig. 2) demonstrates agreement between the two measurement methods. The mean difference (δCr) between CrP and CrC was 0.49 μmol/L (SD: 13.8), with 95% limits of agreement between −27 μmol/L and +28 μmol/L. No significant bias was detectable. The absolute difference in creatinine between the two measurements resulted in a change in AKI status in 3488 (4.4%) of the paired samples.

We also performed a sensitivity analysis comparing the Bland–Altman assessment between CrC and CrP by site (Supplementary Fig. S1), which did not demonstrate any alteration to the described relationship. Additionally, we performed a sensitivity analysis comparing the agreement between the two methods by CrC greater than or less than 250 μmol/L (Supplementary Fig. S2). The sensitivity analysis demonstrated the exact measurements of agreement based on high or low CrC values.

### 3.3 Point-of-care creatinine reliability

The CrP reliability and repeatability were assessed by comparing two samples collected within 1 h from the same patient. The two CrP measurements correlated well with a very high Pearson coefficient of 0.99 (Supplementary Fig. S3). Furthermore, the Bland–Altman plot demonstrates agreement between the two samples, with a mean difference of 0.6 μmol/L (SD: 13.5), with 95% limits of agreement between −27 μmol/L and +28 μmol/L (Supplementary Fig. S4).

## 4 For the cohort

### 4.1 Biochemical variables interacting with point-of-care creatinine measurement

The mixed-effect multivariate linear regression analysis examining the interaction between other biochemical variables and the magnitude of difference between CrC and CrP (the delta creatinine or δCr) is demonstrated in Table 2.

**Table 2:** Multivariate regression of factors affecting the difference in creatinine.

Variable	Coefficient	95% CI^a^	p-value
Lactate (mmol/L)	1.3	1.3, 1.4	<0.001
Ionised calcium (mmol/L)	−0.61	−0.74, −0.49	<0.001
Haemoglobin (g/L)	0.02	0.01, 0.02	<0.001
pH	−0.36	−0.58, −0.15	0.001
PaCO2 (mmHg)	0.00	−0.02, 0.02	0.9
PaO2 (mmHg)	0.00	0.00, 0.00	0.5
Carboxyhemoglobin (%)	0.60	0.36, 0.83	<0.001
Methaemoglobin (%)	−1.8	−2.1, −1.5	<0.001
Potassium (mmol/L)	−2.3	−2.5, −2.1	<0.001
Sodium (mmol/L)	−0.07	−0.10, −0.03	<0.001
Chloride (mmol/L)	0.13	0.10, 0.16	<0.001
Glucose (mmol/L)	0.67	0.63, 0.71	<0.001
Albumin (g/L)	0.12	0.10, 0.14	<0.001
Bilirubin (umol/L)	−0.06	−0.06, −0.06	<0.001
Piperacillin/tazobactam, any	−0.11	−0.37, 0.15	0.4

The difference in creatinine measurements equals laboratory creatinine minus point-of-care creatinine. A positive difference means that the laboratory creatinine was greater than the point-of-care creatinine.

a CI = confidence Interval.

Multiple variables interacted with δCr. A 0.1 unit decrease in pH was associated with a −0.39 μmol/L change in δCr such that CrC was less than CrP (95% CI: -0.61 to −0.17; p < 0.001). Other variables that were independently associated with δCr interacted as follows: a 1-mmol/L increase in potassium resulted in a 2.3-μmol decrease in δCr (95% CI: -2.5 to −2.1; p < 0.001), a 1-mmol/L increase in lactate was associated with a 1.3-μmol increase in δCr (95% CI: 1.3 to 1.4; p < 0.001), a 1-mmol/L increase in glucose was associated with a 0.67-μmol increase in δCr (95% CI: 0.63 to 0.71; p < 0.001), and a 1-μmol/L increase in bilirubin was associated with a −0.06-μmol decrease in δCr (95% CI: -0.06 to −0.06; p < 0.001). In addition, haemoglobin, methaemoglobin, carboxyhemoglobin, ionised calcium, sodium, chloride, and albumin were all associated with a change in δCr. However, the range of possible values for these variables and the magnitude of impact on δCr made the impact of the relationship clinically negligible.

## 5 Discussion

### 5.1 Key findings

In this multicenter study of almost 20,000 critically ill patients and 70,000 paired creatinine measurements, we found similar values between CrC and CrP. The two methods for measuring creatinine were highly correlated, and there was strong agreement between CrC and CrP with a mean difference of less than 0.5 μmol/L. Moreover, this finding was robust, with a strong agreement across all sites and significantly increased creatinine measurements. Furthermore, the absolute difference between measurements was rarely associated with AKI stage differences. In addition, we found multiple biochemical variables interacted with the difference between CrC and CrP. Finally, the clinical significance of the magnitude of difference associated with these independent variables was minimal.

### 5.2 Relationship to literature

The comparison of CrC and CrP was studied in a small single-centre analysis,^8^ which found strong agreement between the two methods of creatinine measurement with a mean difference of 9.6 μmol/L, greater than that found in our study. However, this study included only 82 patients from a single centre. In contrast, our study included almost 20,000 critically ill patients.

A single-centre study of 113 patients from a renal unit^34^ compared creatinine-derived estimated glomerular filtration rate between point-of-care and laboratory measurements and also found a high level of agreement in non–critically ill patients. Another single-centre study of 207 patients in an emergency department compared paired samples of CrC and CrP, demonstrating a mean difference of 1.6 μmol/L. Neither of these studies was large or multicentric and was performed in patients admitted to the ICU; therefore, before our study, their applicability to the critically ill population and their robustness was uncertain.

### 5.3 Implications of study findings

Our research indicates that, in critically ill patients, using arterial blood gas machines for point-of-care creatinine measurements is a viable alternative to the standard creatinine measurement method used in central laboratories. The strong correlation between CrC and CrP suggests that CrP can accurately assess creatinine levels, enabling more frequent evaluations of renal function. Furthermore, the strength of the agreement is increased by the absolute difference in measurements, being unlikely to result in a different severity of AKI. The findings also suggest that, although changes in other blood tests may affect the relationship between CrC and CrP, their impact is not clinically significant. In summary, our findings present the potential for CrP to enable earlier and more frequent evaluations of renal function.

### 5.4 Strengths and limitations

Our study has several strengths. Firstly, it is the largest collection of paired creatinine measurements in the critically ill, which lends a level of robustness close to 200-fold greater than any previous study. Secondly, it was performed across four different ICUs with a diverse patient population and using a machine operated by an estimated 500 plus nurses, lending it a high degree of external validity. Thirdly, it utilised routinely collected data with no patient exclusions, meaning the study's implications apply to all critically ill patients.

We acknowledge some limitations. The agreement between CrC and CrP does not yet imply that POCT can be used to improve the diagnostic and clinical value of creatinine measurements. However, it provides a very robust first step to study arterial blood gas-based frequent creatinine measurements as a way of increasing diagnostic sensitivity and opens the door to such studies. The study findings do not involve any association with clinical outcomes; only the two methods of measurement are comparable in critically ill patients. However, demonstrating that the performance of POCT measurements is robust and not prone to interferences enables such assessment. Finally, the widespread use of CrP is dependent on demonstrating its impact on AKI prediction, incidence, and management, which is an area for future research.

## 6 Conclusion

In a study involving over 70,000 paired creatinine samples, we demonstrated a high level of agreement between CrC and CrP. We found a negligible mean difference of 0.5 μmol/L between paired samples. We also found that the difference between CrC and CrP was independently affected by common serum variables; however, the magnitude of their impact was clinically negligible. Our study opens the door to the use of frequent ABG-machine-based creatinine monitoring in ICU patients as a means of earlier and more sensitive AKI diagnosis in this setting.

## Fundings

This research did not receive any specific grant from funding agencies in the public, commercial, or not-for-profit sectors.

## Data availability statement

Data cannot be shared publicly due to institutional ethics, privacy, and confidentiality regulations. Data released for research under Sect. 280 of the Public Health Act 2005 requires an application to the Director-General of Queensland Health (PHA@health.qld.gov.au).

## Conflict of interest

The authors have no conflicts of interest to declare.

## CRediT authorship contribution statement

The study conception and design (KW, RB); data acquisition (KW); analysis (KW); interpretation of data (all authors); article draughting (KW, RB), article revision for important intellectual content (all authors); final approval of the version submitted for publication (all authors); agreement to be accountable for all aspects of the work in ensuring that questions related to the accuracy or integrity of any part of the work are appropriately investigated and resolved (KW, RB).
