# Supplementary material for: Point-of-care creatinine vs. central laboratory creatinine in the critically ill
Source: Crit Care Resusc. 2024 Aug 5;26(3):198–203. doi: 10.1016/j.ccrj.2024.07.002 (PMC11440060; doi:10.1016/j.ccrj.2024.07.002)
Supplement: Multimedia component 1 [file mmc1.docx]

**Point of care creatinine vs. central laboratory serum creatinine in the critically ill**

**Online Supplement**

| *Table S1. Creation of Diagnostic Groups* | |
| --- | --- |
| **Category** | **APACHE-III J Diagnostic Codes** |
| Cardiovascular | 101-111  1202-1213 |
| Respiratory | 201-213,  1301-1304 |
| Gastrointestinal | 301-313  1401-1413 |
| Neurological | 401-410,  1501-1506 |
| Sepsis | 501-504 |
| Trauma | 601- 605,  1601-1605 |
| Metabolic | 701-704,2201 |
| Haematological | 801,802,2101 |
| Genitourinary | 901-903,1701-1705,  1801-1803 |
| Musculoskeletal | 1101,1102 |
| Other | ‘other codes’ |
| APACHE = Acute Physiology and Chronic Health Evaluation | |
|  |  |

*Table S2. Calculation of Charlson Co-morbidity Index*

| **Category** | **ICD-10 Codes** | **Weight** |
| --- | --- | --- |
| Ischaemic Heart Disease | I21, I22, I252 | 1 |
| Congestive Heart Failure | I50 | 1 |
| Peripheral vascular disease | I71, I790, I739, R02, Z958, Z959 | 1 |
| Cerebral vascular disease | I60, I61, I62, I63, I65, I66, G450, G451, G452, G458, G459, G46, I64, G454, I670, I671, I672, I674, I675, I676, I677 I678, I679, I681, I682, I688, I69 | 1 |
| Dementia | F00, F01, F02, F051 | 1 |
| Chronic pulmonary disease | J40, J41, J42, J44, J43, J45, J46, J47, J67, J44, J60, J61, J62, J63, J66, J64, J65 | 1 |
| Connective tissue disease | M32, M34, M332, M053, M058, M059, M060, M063, M069, M050, M052, M051, M353 | 1 |
| Peptic ulcer disease | K25, K26, K27, K28 | 1 |
| Mild liver disease | K702, K703, K73, K717, K740, K742, K746, K743, K744, K745 | 1 |
| Moderate-severe liver disease | K729, K766, K767, K721 | 3 |
| Diabetes | E109, E119, E139, E149, E101, E111, E131, E141, E105, E115, E135, E145 | 1 |
| Diabetes with complications | E102, E112, E132, E142 E103, E113, E133, E143 E104, E114, E134, E144 | 2 |
| Paraplegia | G81 G041, G820, G821, G822 | 2 |
| Chronic kidney disease | N03, N052, N053, N054, N055, N056, N072, N073, N074, N01, N18, N19, N25 | 2 |
| Localised cancer | C0, C1, C2, C3, C40, C41, C43, C45, C46, C47, C48, C49, C5, C6, C70, C71, C72, C73, C74, C75, C76, C80, C81, C82, C83, C84, C85, C883, C887, C889, C900, C901, C91, C92, C93, C940, C941, C942, C943, C9451, C947, C95, C96 | 2 |
| Metastatic cancer | C77, C78, C79, C80 | 3 |
| HIV | B20, B21, B22, B23, B24 | 6 |

Table S3. Multivariate Regression of Factors affecting the Difference in Creatinine with one measurement per patient

| Variable | **Coefficient** | **95% CI**^1^ | **p-value** |
| --- | --- | --- | --- |
| Lactate (mmol/L) | 1.2 | 1.1, 1.3 | <0.001 |
| Ionised Calcium (mmol/L) | -0.03 | -0.24, 0.19 | 0.8 |
| Hemoglobin (g/L) | 0.02 | 0.01, 0.03 | <0.001 |
| pH | -0.54 | -0.87, -0.20 | 0.002 |
| PaCO2 (mmHg) | -0.04 | -0.07, -0.01 | 0.007 |
| PaO2 (mmHg) | -0.01 | -0.01, 0.00 | <0.001 |
| SaO2 (%) | 0.03 | -0.02, 0.07 | 0.2 |
| Carboxyhemoglobin (%) | 0.88 | 0.56, 1.2 | <0.001 |
| Methemoglobin (%) | -2.1 | -2.5, -1.7 | <0.001 |
| Potassium (mmol/L) | -1.5 | -1.8, -1.2 | <0.001 |
| Sodium (mmol/L) | -0.07 | -0.12, -0.01 | 0.019 |
| Chloride (mmol/L) | 0.10 | 0.05, 0.15 | <0.001 |
| Glucose (mmol/L) | 0.83 | 0.77, 0.88 | <0.001 |
| Albumin (g/L) | 0.08 | 0.05, 0.11 | <0.001 |
| Bilirubin (µmol/L) | -0.05 | -0.06, -0.05 | <0.001 |
| Piperacillin/Tazobactam, any | 0.16 | -0.33, 0.65 | 0.5 |
| ^1^CI = Confidence Interval | | | |
| The difference in creatinine measurements equals laboratory creatinine minus point-of-care creatinine. A positive difference means that the laboratory creatinine was greater than the point-of-care creatinine. | | | |

| **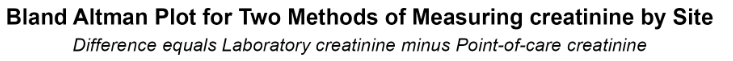** | |
| --- | --- |
| **Site A** | **Site B** |
| **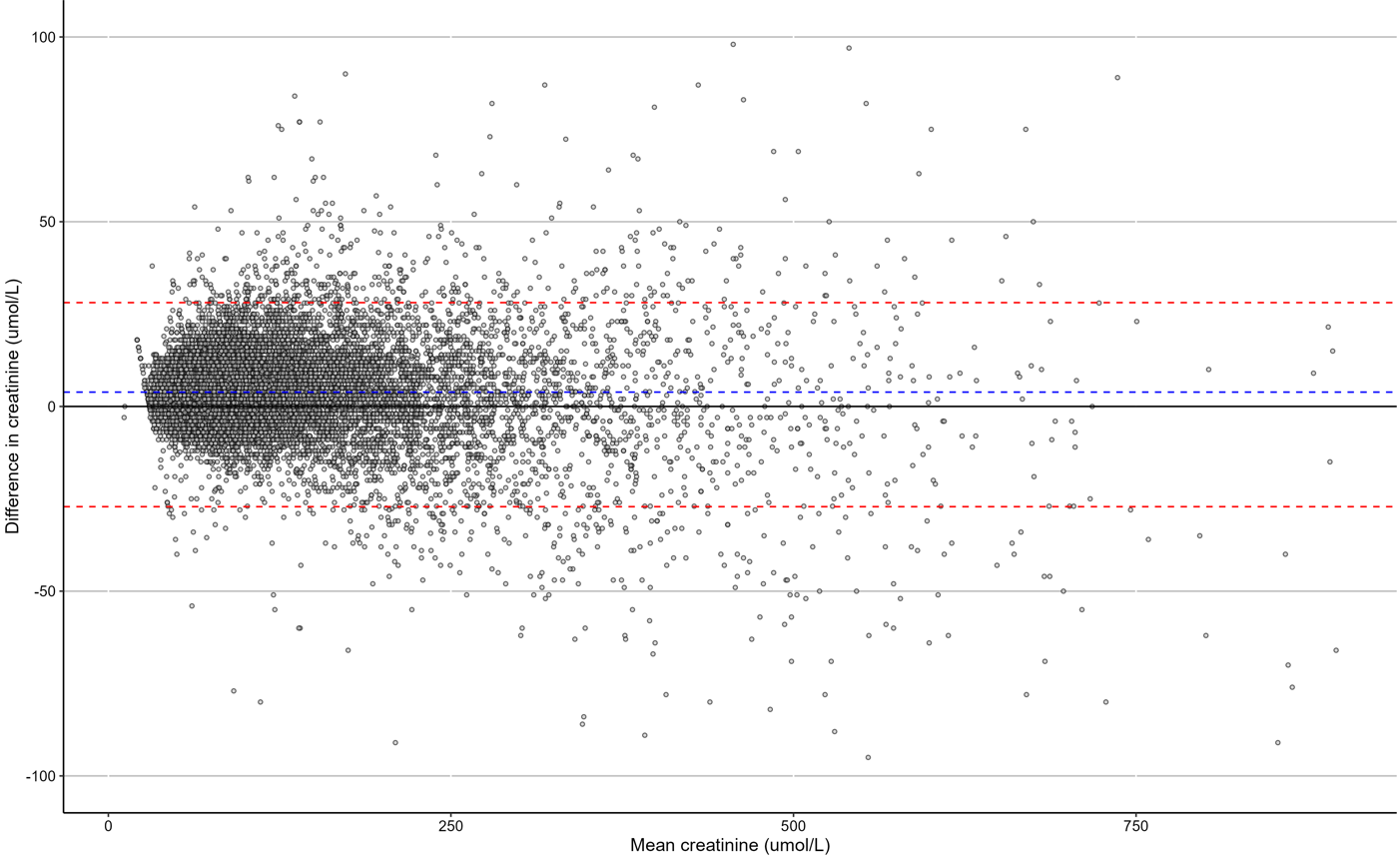** | **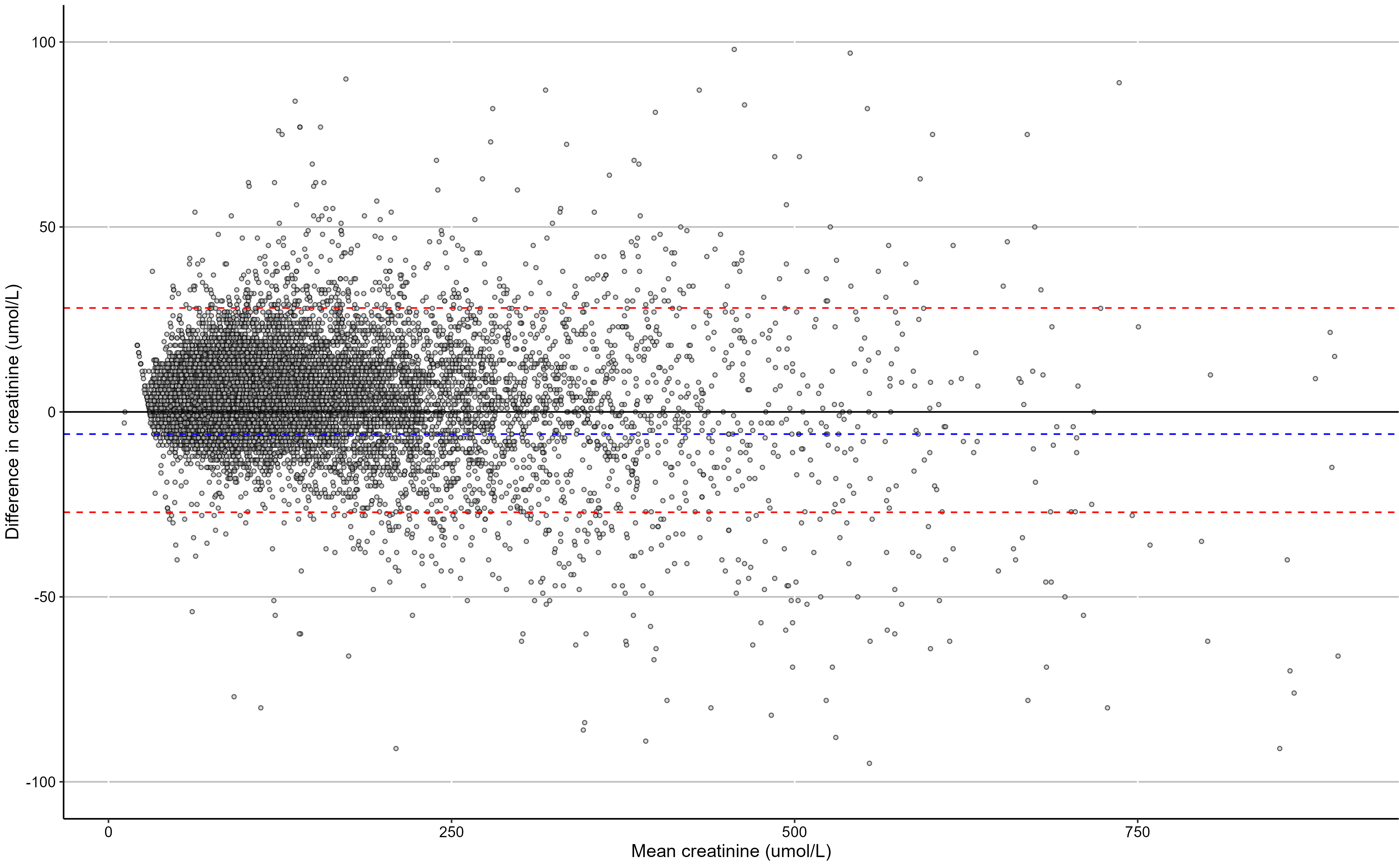** |
| **Site C** | **Site D** |
| **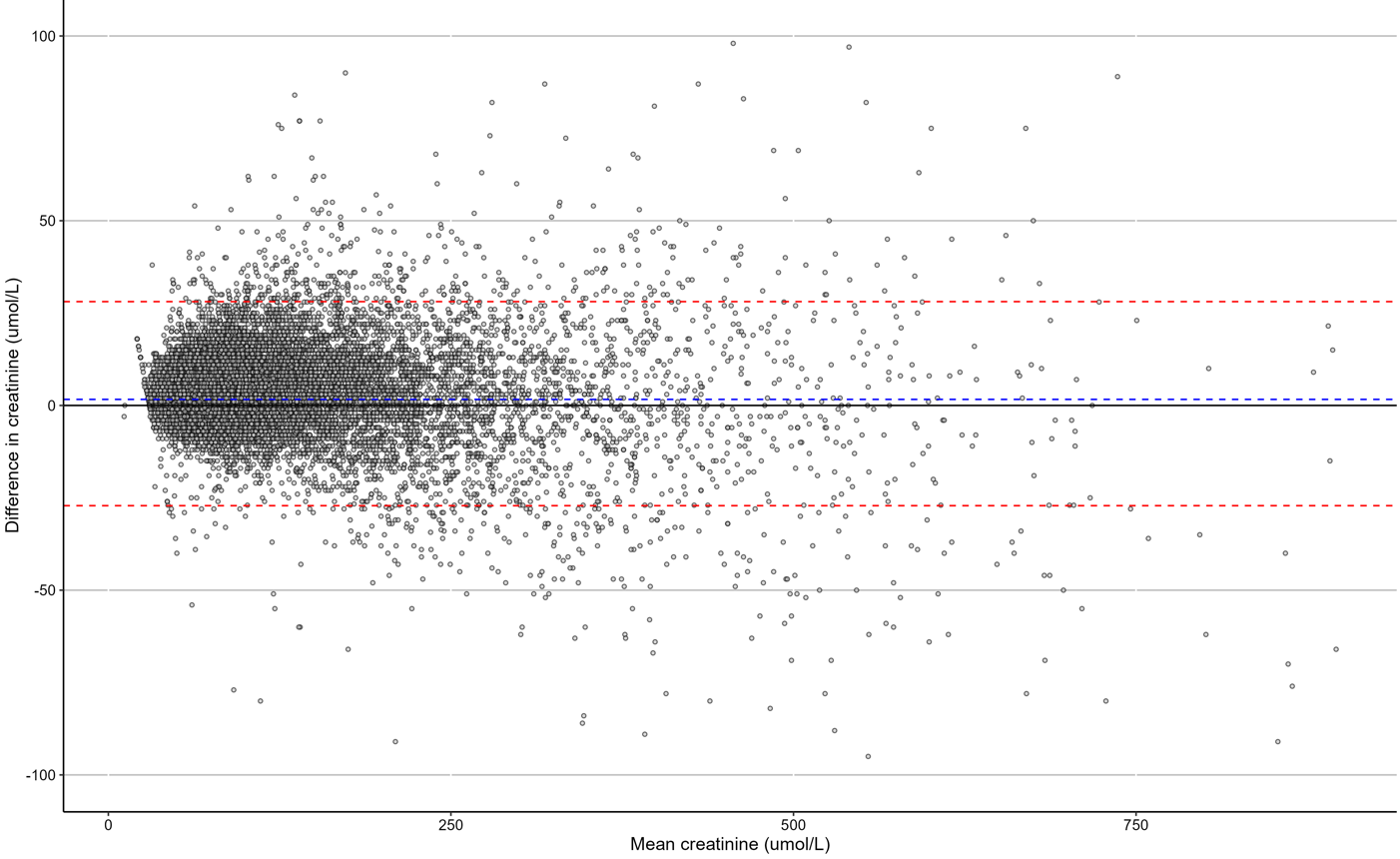** | **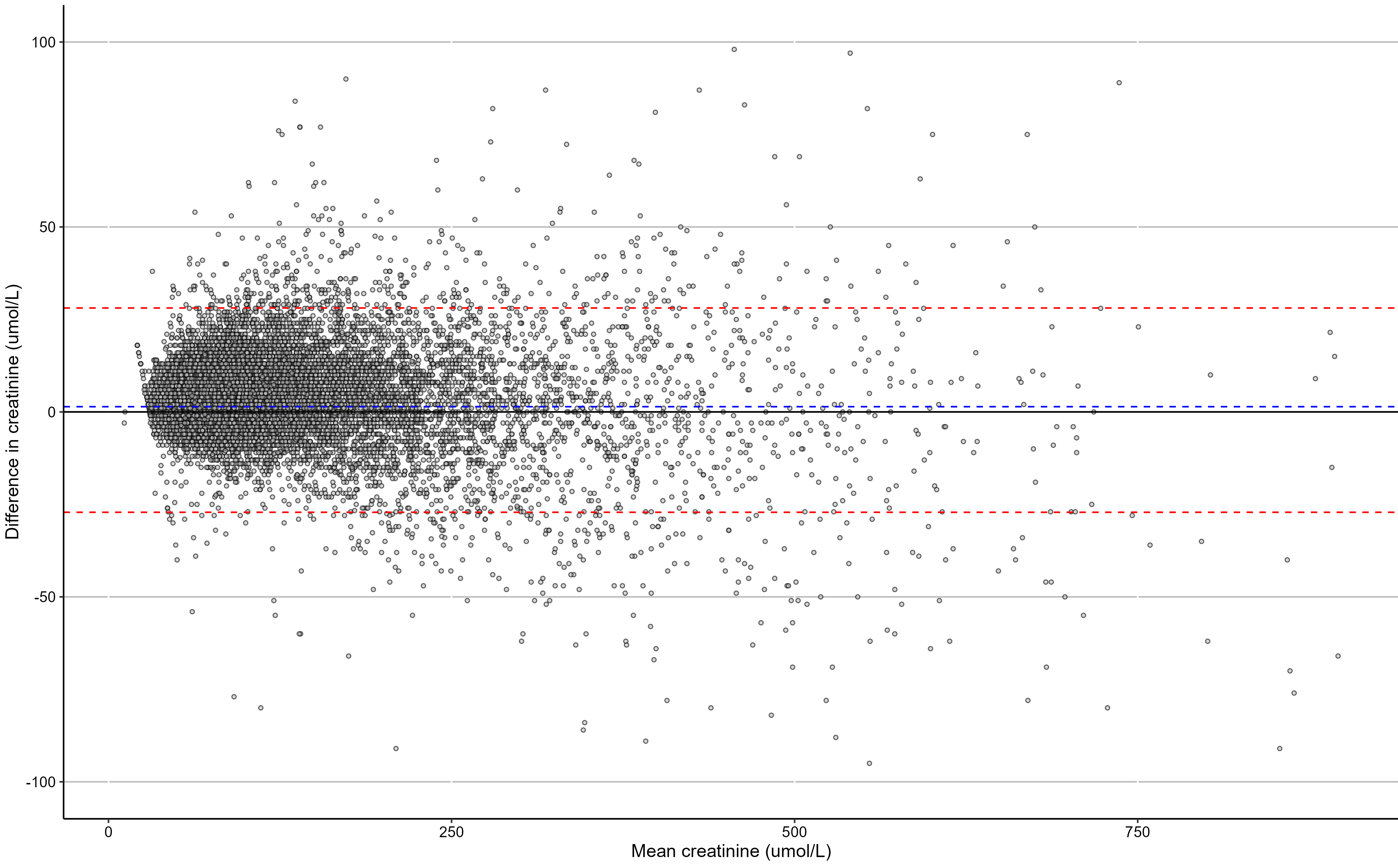** |

**Figure S1**: Bland Altman Plot for Two Methods of Measuring creatinine by level of laboratory creatinine by site


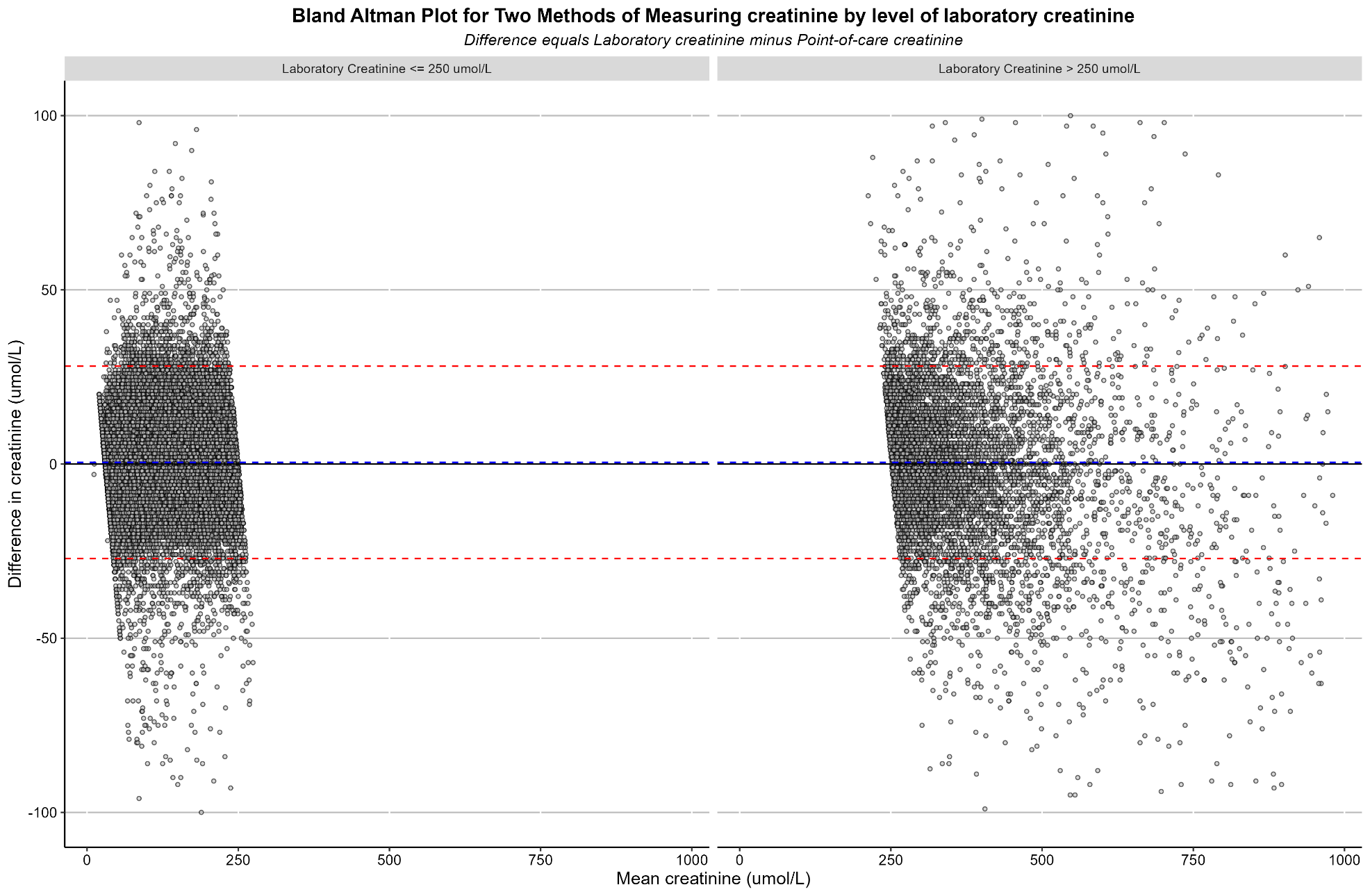


**Figure S2**: Bland Altman Plot for Two Methods of Measuring creatinine by level of laboratory creatinine


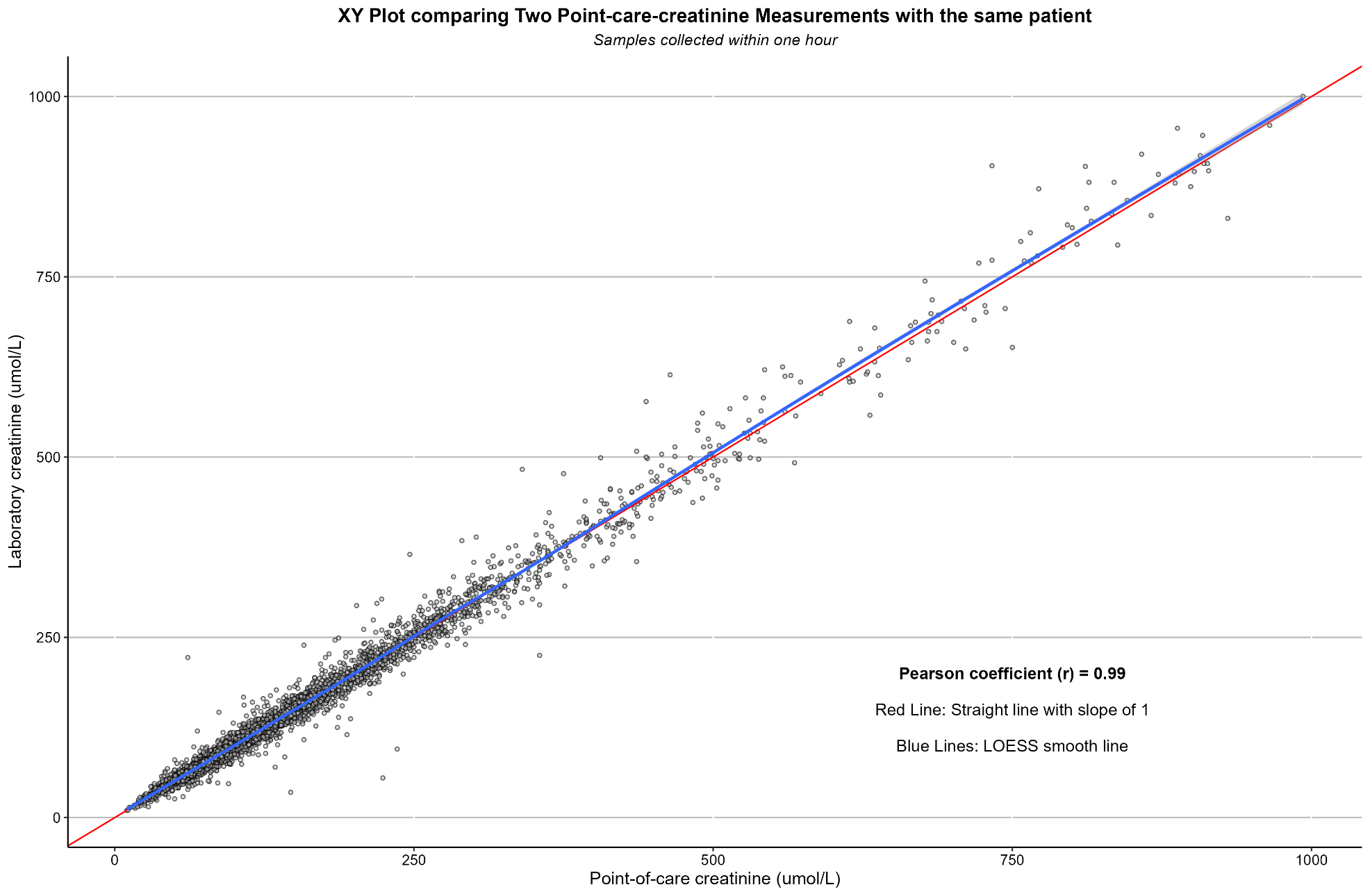


**Figure S3**: XY Plot comparing Two Point-care-creatinine Measurements with the same the patient

**
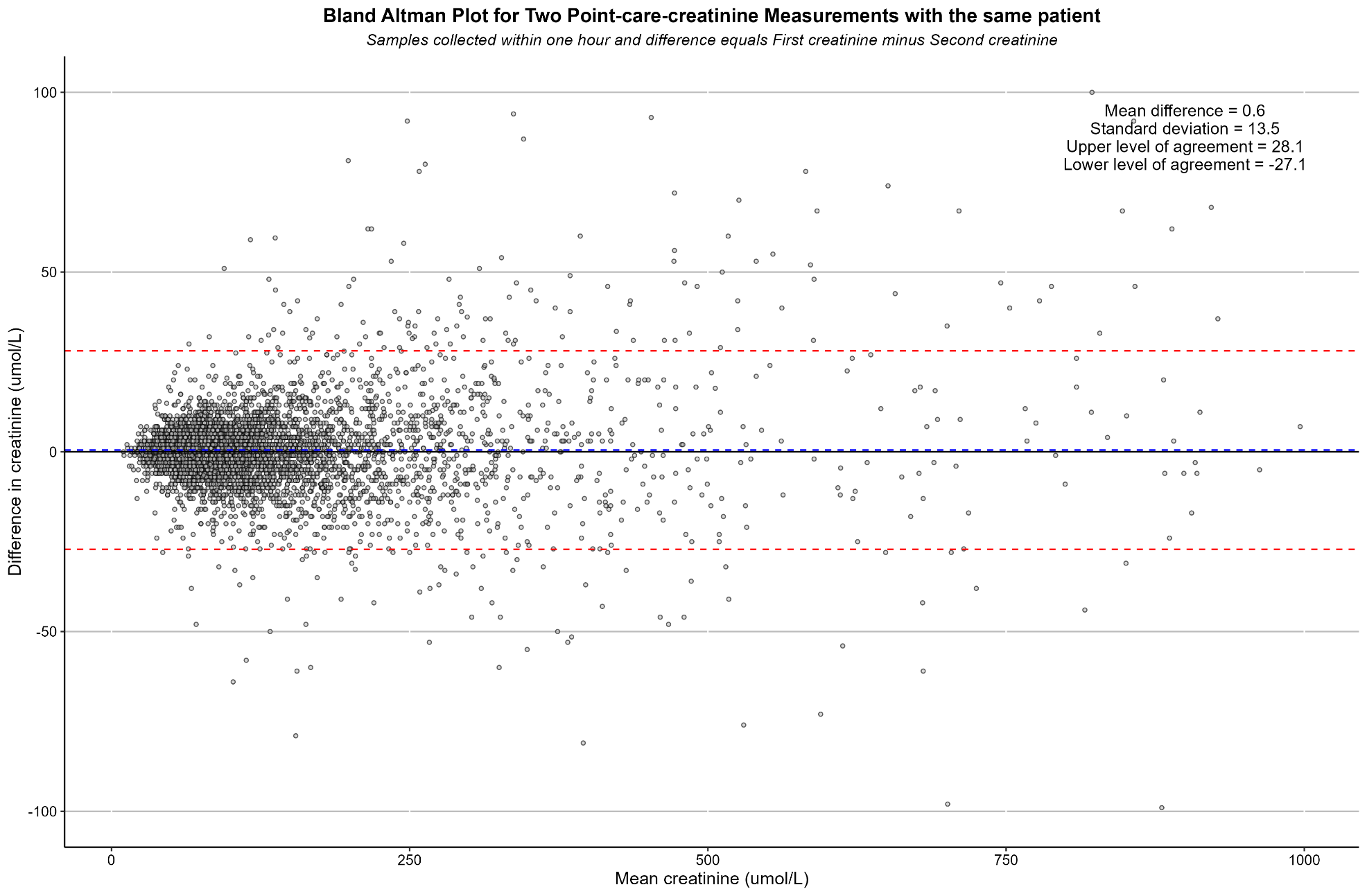
**

**Figure S4**: Bland Altman Plot for Two Point-care-creatinine Measurements with the same patient
